# Multi-scale and deeply supervised network for image splicing localization

**DOI:** 10.3389/frai.2025.1655073

**Published:** 2025-09-25

**Authors:** Sheng Qin, Ce Liang, Yuling Luo, Junxiu Liu, Qiang Fu, Xue Ouyang

**Affiliations:** ^1^Guangxi Key Lab of Brain-Inspired Computing and Intelligent Chips, School of Electronic and Information Engineering, Guangxi Normal University, Guilin, China; ^2^Key Laboratory of Nonlinear Circuits and Optical Communications (Guangxi Normal University), Education Department of Guangxi Zhuang Autonomous Region, Guilin, China

**Keywords:** image forensics, image splicing, multi-scale, encoder–decoder, deep learning

## Abstract

When maliciously tampered images are disseminated in the media, they can potentially cause adverse effects and even jeopardize national security. Therefore, it is necessary to investigate effective methods to detect tampered images. As a challenging task, the localization of image splicing tampering investigates whether an image contains tampered regions spliced from another image. Given the lack of global information interactions in existing methods, a multi-scale, deeply supervised image splicing tampering localization network is proposed. The proposed network is based on an encoder–decoder architecture, where the decoder uses different levels of feature maps to supervise the locations of splicing, enabling pixel-wise prediction of tampered regions. Moreover, a multi-scale feature extraction module is utilized between the encoder and decoder, which expands the global view of the network, thereby enabling more effective differentiation between tampered and non-tampered regions. F1 scores of 0.891 and 0.864 were achieved using the CASIA and COLUMB datasets, respectively; and the proposed model was able to accurately locate tampered regions.

## 1 Introduction

Digital media has become the main form of information exchange. The expanding social media networks and various fields such as military, legal, political, medical, education, and business all depend on digital media to accomplish various crucial tasks. Compared to text, images provide a more intuitive way to convey information. In the past, many people had great confidence in the information conveyed by images. However, this credibility is constantly decreasing in the current time. A large amount of image data on the Internet has been tampered with and disseminated through various digital media platforms, as people can easily learn to use powerful image editing software such as Photoshop, CorelDraw, and GIMP to edit or manipulate images (Farid, 2009; Carvalho et al., 2013; Liao et al., 2020). Among the most common types of image tampering is image splicing tampering, which combines different parts of two or more images to create a new one. Image splicing tampering has the potential to be used for a variety of purposes, such as creating fake news and publicity, leading to serious security concerns. Therefore, the detection of image splicing has become increasingly important in the field of digital forensics, and image splicing detection and localization have attracted widespread attention from researchers in recent years.

The detection of image splicing (He et al., 2012; Zhao et al., 2015; El-Alfy and Qureshi, 2015) aims to determine whether an image has been spliced, which is a problem of image-level classification. However, the spliced regions of the image cannot be located by them. The spliced regions of the image often convey more splicing information and are more in line with practical needs, but the task of locating these regions poses a greater challenge. The task of image splicing tampering location is more challenging and important than splicing detection.

Traditional methods for locating tampered regions in image splicing mainly rely on comparing features between different regions of the image. There are three types of traditional methods based on manual design features for image splicing tampering localization: those based on inconsistent blur types, those based on inconsistent noise levels, and those based on inconsistent lighting conditions. In methods based on the inconsistency of blur types, Kakar, Sudha, and Ser proposed a method that uses differences in motion blur to locate the image splicing region (Kakar et al., 2011). Image tampering causes the motion blur in the tampered region to be inconsistent with the blur in the rest of the image. Using this property, the regions of image tampering can be located. A framework for detecting and locating image splicing was proposed by Bahrami et al. (2013), based on inconsistencies between blur and depth information in the image. The splicing region is identified by classifying image blocks with different levels of blur. Image splicing tampering can also cause differences in noise distribution. Some researchers have conducted studies on image splicing detection and localization algorithms based on the inconsistency of noise in tampered images. The approach of Mahdian and Saic (2009) divides the image into non-overlapping image blocks and uses a median-based method to evaluate the noise standard deviation of each block to delineate the tampered regions on the image. Typically, shadow and lighting inconsistencies exist in images used for splicing manipulation due to different shooting angles. Based on this property, a scheme for image splicing tampering detection based on shadow brightness inconsistencies is proposed (Liu et al., 2011). It extracts shadow boundaries and half-shadow regions in the image and determines the tampered regions by evaluating the consistency of the shadow mask values of the shadows in the image. However, the method fails when the shadows of the tampered region are consistent with the shadows of the source region. This shows that these traditional methods for tampering detection and localization are often only applicable to images under some specific conditions, and the generality of the algorithm is poor.

Given the successful application of deep learning techniques in various computer vision tasks, research has begun to explore leveraging the adaptive nature of deep learning models to automatically extract splicing tampering features from images (Su et al., 2025; Jiang et al., 2025; Zhang et al., 2024; Hou et al., 2024). Inspired by the use of full convolutional networks (Long et al., 2015) for semantic segmentation tasks, a multi-task network framework (Salloum et al., 2018) based on edge enhancement is proposed for achieving pixel-level image splicing tampering localization. The method extracts the tampering features of the image using VGG-16 and subsequently uses full convolution to form two decoding branches. One decoding branch predicts the image tampered regions, and the other one is used to predict the edges of the tampered region. In Xiao et al.'s study (Xiao et al., 2020), a cascade-based convolutional neural network (CNN) was proposed for image splicing tampering localization, which consists of two main parts. One part is a CNN from coarse to fine, and this part first roughly learns the difference between tampered and untampered regions in the image, with a particular focus on the edges. The other part is an adaptive clustering algorithm, which is used to get the final results. The approach by Bi et al. (2019) is also based on the idea of image segmentation to design a novel network with encoding modules consisting of residual propagation and residual feedback. The network is able to effectively distinguish between untampered and tampered regions. The approach by Wei et al. (2021) devises a clever strategy to control the size of the local perceptual field of each building block, based on the work of Bi et al. (2019) and uses BAM attention (Park et al., 2018). However, its ability to capture global contextual information in the image is still limited. Most of the existing splicing tampering localization methods can only provide rough locations of tampered regions or detect localization unsatisfactorily, and the connection between global features is not considered.

To demonstrate the architectural innovations of the proposed model, a direct comparison was made with representative two-branch encoder–decoder frameworks, such as those developed by Zhu et al. (2022) and Luo et al. (2024), from which the proposed architecture distinguishes itself in three fundamental aspects. Firstly, unlike prior methods that assign separate branches to different tasks (e.g., region and edge prediction), the proposed model employs a deep supervision strategy where all decoder side outputs contribute to the single primary task of predicting tampered region masks. These multi-scale outputs are subsequently fused to generate a more refined and accurate result, thereby concentrating the network's full capacity on a unified objective. Secondly, the model is built upon the U^2^-Net backbone, a nested U-structure where each stage (Residual U-block (RSU) module) is itself a U-Net-like architecture. This design facilitates significantly richer multi-scale feature extraction and a more powerful capture of intra- and inter-scale contextual information than standard VGG-based encoders. Finally, to overcome the critical limitation of insufficient global context modeling in purely convolutional approaches, this study integrates a multi-scale Transformer module between the encoder and decoder. This module utilizes self-attention mechanisms to explicitly model long-range dependencies across the entire feature map, providing a comprehensive global perspective that is otherwise difficult to achieve. In this study, a multi-scale, deeply supervised image splicing tampering localization network is proposed to accurately locate the tampered region of the spliced image at the pixel level. The proposed network employs a fully convolutional encoder–decoder architecture. In the decoder, a multi-resolution feature map is utilized from low to high resolution to perform supervision for the localization of the tampered region, which enhances the use of shallow information and enables pixel-wise prediction of the tampered region. Moreover, a multi-scale feature extraction module is utilized between the encoder and decoder to extend the network's global view and more effectively discover the features of the tampered region, thereby detection accuracy is improved. Experiments are performed on two standard tampered datasets, and ultimately, it is evident that the proposed model achieves good localization performance.

In summary, the main contributions are as follows: (1) a multi-scale, deeply supervised image splicing localization network is proposed, which can effectively locate spliced regions in the image. (2) A multi-scale feature extraction module is introduced to better capture the features of spliced regions. (3) The effectiveness of the proposed model is verified on the CASIA (Dong et al., 2013) and COLUMB (Paszke et al., 2017) datasets, where the F1 score reached 0.891 and 0.864, respectively.

The rest of the article is organized as follows: Section 2 briefly introduces some prerequisite knowledge. Section 3 provides a detailed description of the model structure. Section 4 reports the experimental results and experimental analysis. Finally, Section 5 presents the conclusion.

## 2 Preliminaries

**Figure 1:**
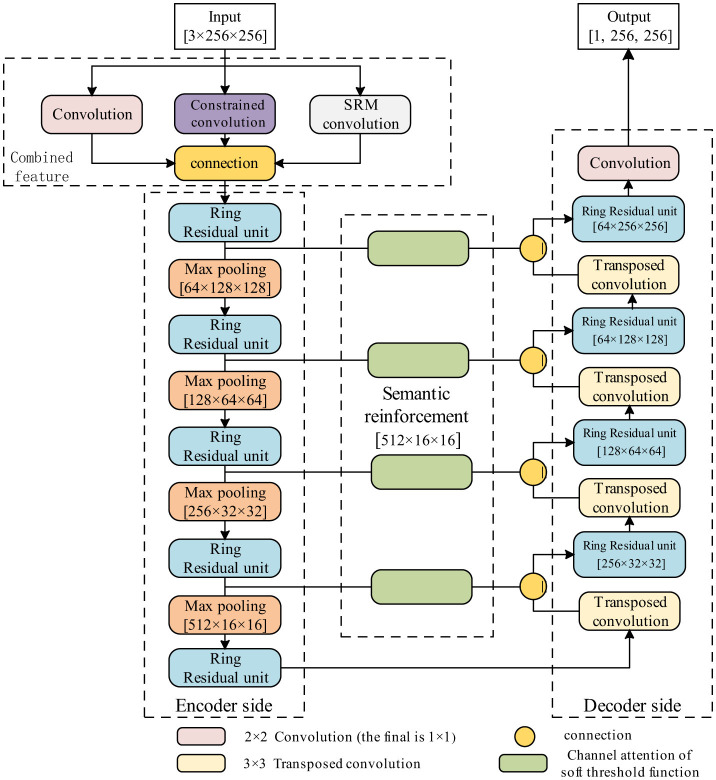
A flowchart of the U-Net-like encoder–decoder architecture for image splicing localization.

Image tampering localization can be considered a complex image segmentation task. Therefore, it is possible to apply convolutional neural network-based image segmentation methods for the localization of tampered regions in images. U-Net was initially proposed for medical image segmentation (Ronneberger et al., 2015). Due to its excellent performance, U-Net has become not only a popular network in the field of medical image segmentation but also has led to the emergence of various networks based on U-Net. These networks are widely used in different fields, such as satellite image segmentation (Soni et al., 2020) and industrial defect detection (Cui et al., 2023). Similarly, U-Net-based improved networks have also been used for image tampering localization (Shi et al., 2020; Bi et al., 2019). Specifically, U-Net is an end-to-end network designed for image segmentation tasks, built upon the foundation of a fully convolutional neural network. Adopting the classic encoder–decoder architecture, U-Net is symmetrical from left to right, comprising three key components: The contracting path (encoder) on the left, the expanding path (decoder) on the right, and the skip connection. Figure 1 illustrates a concrete example of such a U-Net-like encoder–decoder framework applied to the task of image splicing localization. In this architecture, the initial input image passes through a combined features module, which often includes parallel convolutional layers such as a standard convolution and spatial rich model (SRM) to extract rich low-level features. The encoder path, composed of a series of ringed residual units and max-pooling layers, is responsible for progressive downsampling to capture deep semantic information. Subsequently, the decoder path performs upsampling via deconvolution and fuses shallow features from the corresponding encoder levels using skip connections, thereby precisely recovering details and spatial resolution. A semantic enhancement module bridges the encoder and decoder paths. Finally, an output convolutional layer generates the pixel-level localization mask. The contracting path consists of CNNs for extracting the features of the input. In addition, 2 × 2 max-pooling layers are used in the contracting path to downsample the feature maps, and the number of feature channels is doubled after each downsampling. The expanding path upsamples the high-dimensional feature maps through transpose convolution, restores feature map resolution, and reduces the count of feature map channels by half. In the end, the feature map is mapped by a 1 × 1 convolution to yield the final segmentation result, and the segmentation mask is output. Although there are some discriminative features that can be extracted from the dataset using U-Net, they are more likely to be less discriminative and not sufficient to localize the tampered region.

Numerous CNNs have been successfully used for various fields, such as image classification (Krizhevsky et al., 2012), object detection (Ren et al., 2017), and segmentation (Ronneberger et al., 2015). Although the CNN is advantageous in local feature extraction, there are difficulties in global feature extraction. The Transformer (Vaswani et al., 2017), due to its powerful ability to model long-term contextual information in natural language processing tasks, has made a deep impression on people. In recent years, some researchers have introduced it into computer vision tasks. The approach by Dosovitskiy et al. (2020) applies the standard Transformer to images. Specifically, the image is first segmented into small blocks and then fed into the Transformer through positional encoding. The global information of the input image is modeled in the Transformer using self-attention and a multi-layer perceptron, with excellent results in image recognition tasks. In the approach by Carion et al. (2020), the Transformer is applied for object detection. The CNN is first used to extract the feature maps, then the extracted feature maps are position encoded and fed to the encoder and decoder consisting of the Transformer. Finally, the object is obtained using prediction heads. Given that image splicing tampering involves a broad range of contextual information, a more globally aware approach is needed.

A noteworthy trend in image tampering localization research is the adoption of multi-stream or multi-modal network architecture, which aims to extract complementary tampering features from different information dimensions. A recent model is the two-branch network proposed by Luo et al. (2024). The core idea of their model is to process two different information streams from the same image in parallel: one branch (the RGB stream) learns content and tampering features directly from the image's spatial domain, while the other branch (the frequency stream) uses the Frequency-Aware Decomposition (FAD) module to specifically extract frequency-domain features that can reveal subtle forgery artifacts. Finally, the feature maps from both branches are effectively integrated using the Convolutional Block Attention Module (CBAM) to enhance focus on the tampered regions. The rationale behind this approach is that information from the spatial and frequency domains can complement each other to achieve more robust tampering localization.

The single-backbone, multi-scale framework proposed in this study differs substantially from the methodologies described above. This method does not focus on fusing inputs from different modalities. Instead, it aims to extract deeper and broader contextual features from a single RGB information stream by leveraging the deeply nested U^2^-Net architecture and an innovative multi-scale Transformer module. It improves localization accuracy by enhancing the model's global perspective and the richness of its feature representations, whereas the model proposed by Luo et al. (2024) represents another effective technical path that enhances robustness through the fusion of heterogeneous information sources.

## 3 Proposed model

This model introduces a hierarchical, two-level multi-scale feature fusion strategy. This strategy creates a more comprehensive understanding of image content than existing single-level approaches. The proposed model has two distinct levels. At the local level, the model uses a U^2^-Net backbone. This nested U-Net structure has Residual U-blocks (RSU). They are designed to capture rich, multi-scale contextual information within each stage of the encoder and decoder. This provides a detailed feature representation before any global analysis. At the global level, the proposed model introduces a multi-scale Transformer module between the encoder and decoder. This module uses a self-attention mechanism across various patch scales (e.g., 16x16, 8x8, 4x4, and 2x2) to capture long-range dependencies. The main innovation is the synergy between these two levels. The U^2^-Net generates a detailed, locally-aware feature set. The Transformer then contextualizes these features globally. This holistic approach ensures effective modeling of both fine-grained tampering artifacts and large-scale semantic inconsistencies.

The hierarchical architecture offers distinct advantages over state-of-the-art models. While approaches such as MCNL-Net integrate CNNs with attention mechanisms (e.g., BAM), and Mobile-Pspnet prioritizes lightweight design, the proposed model fundamentally enhances global contextual modeling through its two-level multi-scale strategy. This design synergistically captures both core tampered regions and subtle boundary artifacts, enabling comprehensive localization of spliced content. Empirical validation of these advantages (including comparative F1 scores and recall rates against benchmarks) is systematically presented in Section 4.3.2 (Quantitative Results) and visually corroborated using heatmap analyses in Section 4.3.3 (Qualitative Results).

This model is inspired by M2TR's multi-scale attention module. However, the model innovatively integrates this concept into a nested, high-resolution segmentation backbone such as U^2^-Net. This creates specialized architecture for image splicing localization. Unlike M2TR, which is a general deepfake detection framework, the proposed model is tailored to address the unique challenges of image splicing localization.

**Figure 2:**
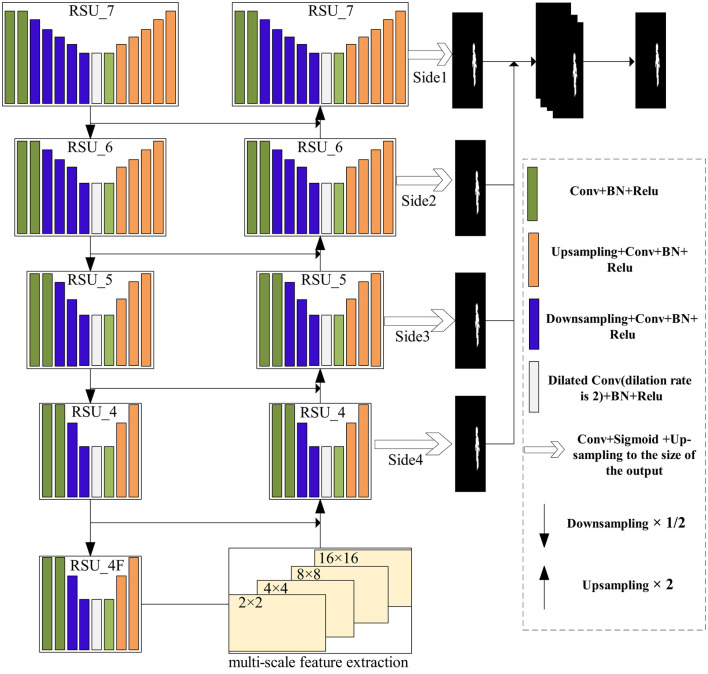
The overall framework of the network.

As shown in Figure 2, a network is proposed with multi-scale and deep supervision characteristics, which can locate the tampered region in spliced tampered images. Inspired by the outstanding performance of U^2^-Net in salient object detection (Qin et al., 2020), in the context of image tampering detection, the difference between the tampered and untampered regions in an image can be regarded as a saliency task, and the tampered region in the image can be segmented. Therefore, theoretically, applying U^2^-Net to image tampering detection is feasible. The fully convolutional network structure of U^2^-Net also enables the model to obtain good segmentation results without complicated post-processing. Moreover, a multi-scale feature extraction module is employed during the transition from network encoding to decoding. This module operates on feature maps of different spatial sizes to detect local inconsistencies in tampered regions of various size levels. Afterward, the decoder learns from the feature maps at low resolution and uses deep supervision during training to enable the network to be more fully trained. Finally, the network can predict the tampered region on a per-pixel basis. Due to its structure, the network can locate spliced regions in an image from a global perspective. Therefore, it can achieve good results in detecting image splicing in most cases. In the following sections, the construction of the proposed model is described in detail.

### 3.1 U^2^-Net-based backbone network architecture

According to the analysis, it was found that the U^2^-Net-based network is suitable for achieving the localization of tampered regions in image splicing at the pixel level. U^2^-Net is a network structure proposed on the basis of U-Net (Ronneberger et al., 2015), which is also an encoding–decoding network structure. RSU is used for network extraction features. Each RSU is constructed based on the U-Net network. It can be said that U^2^-Net is a nested network based on U-Net, where the residual block and the overall network are shaped like a U. Therefore, the network structure is a nested U-Net, which strengthens the learning capability of the model and increases the learning of multiple scales to obtain good localization results.

**Figure 3:**
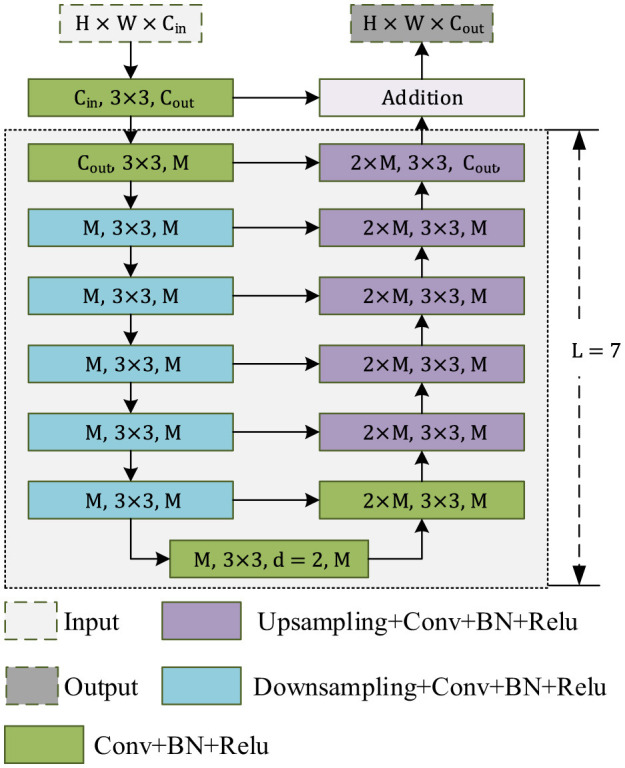
Structure of the residual U-block, L is the layer parameter.

The RSU module is used as the main encoding module of the network. As shown in Figure 3, RSU_L is composed of an input layer that gathers local features and conversion channels, a U-shaped network structure that extracts and encodes contextual information, and an output layer that combines the input layer and middle layer. Similar to U-Net, the left half of the structure is the encoding stage, which extracts features using the 3 × 3 convolution and increases the receptive field by downsampling. The right half is the decoding structure, which upsamples features to a high-resolution feature map and cascades the feature map at the encoding and decoding using a skip connection. The value of L is typically set according to the size of the feature map. The larger the size of the feature map, the larger the L used to obtain more information. Therefore, in the encoding stage, the value of L generally decreases from the upper layer to the lower layer. In Figure 3, when L is seven, the input channel number is *C*_*in*_, the middle channel number is *M*, and the output channel number is *C*_*out*_. The feature map is first transformed by the 3 × 3 convolution for channel number transformation, then it enters the middle layer of the entire structure. The 3 × 3 convolution and 1/2 downsampling are used at the left stage of the middle layer. Since *L* = 7, the left stage of the middle layer uses seven layers of cascaded convolution with downsampling at each layer. The seventh layer uses dilated convolution. Then, the feature map is sent to the right side of the structure. The feature maps of each layer on the right side and the corresponding layer on the left side are superimposed by channels, and the 3 × 3 convolution and upsampling are performed. Finally, the output of the middle layer and the input of the middle layer are added to obtain the output of an RSU. This is the workflow of the RSU.

The backbone network based on U^2^-Net is mainly composed of the following parts: The five-layer encoder, the four-layer decoder, and the binary map for tampered region localization obtained through multi-level supervision. As shown in Figure 2, the network uses RSU_7, RSU_6, RSU_5, RSU_4, and RSU_4F for the encoding stage, with each layer connected through the 2 × 2 max-pooling layer. Therefore, the size of each layer's feature map is reduced by half (except for the last layer of the encoder, which does not use pooling). Specifically, RSU_7 has an input channel of 3, a middle channel of 32, and an output channel of 64. RSU_6 has an input channel of 64, a middle channel of 32, and an output channel of 128. RSU_5 has an input channel of 128, a middle channel of 32, and an output channel of 256. The last two RSU_4 modules (RSU_4 and RSU_4F) have input channels of 256 and 512, middle channels of 128 and 256, and output channels of 512, respectively. The 16 × 16 × 512 feature maps are obtained in the encoding stage. For the upsample process on the right side of the network, each stage uses an RSU that corresponds to the structure on the left encoding side. However, since the output of the decoder's RSU is concatenated with the corresponding feature map from the left side, the input channel, middle channel, and output channel settings of the RSU are different from those on the right side. Specifically, the input channel of RSU_4 in the decoder is 1,024, the middle channel is 128, and the output channel is 256. RSU_5 has an input channel of 512, a middle channel of 64, and an output channel of 128. RSU_6 has an input channel of 256, a middle channel of 32, and an output channel of 64. RSU_7 has an input channel of 128, a middle channel of 16, and an output channel of 64.

In this network, deep supervision refers to a training mechanism where multiple intermediate feature maps of the decoder are supervised via loss functions. As shown in Figure 2, the feature maps output by each RSU in the decoder (e.g., side outputs Side1–Side4) are directly compared to the ground-truth mask to calculate auxiliary losses, which enhance gradient flow and improve shallow feature extraction. On the other hand, side supervision is the supervision applied to individual side paths within the decoder. Each side path corresponds to a specific resolution level (e.g., Side1 for high-resolution features), and its loss $l_{side}^{n}$ is weighted in the total loss (Equation 1). This multi-level approach enables pixel-level prediction of tampered regions. The overall framework, as depicted in Figure 1, presents the deep supervision path (decoder branch) and side supervision (specific path outputs). Nested supervision is a training mechanism that applies separate auxiliary loss functions to each side path, forming a multi-scale constraint system. This “nested” design ensures that supervisory signals propagate only within specific paths, avoiding inter-path interference.

Each side path (Side 1–Side 4 in Figure 2) corresponds to RSU (Figure 3) in the decoder. The RSU's U-shaped architecture enables multi-scale feature extraction within a single path. First, the input layer performs feature transformation through a convolutional layer (filter size 3 × 3), projecting the input feature maps from Cin to Cout. Then, the intermediate layer consists of downsampling (with *L* = 7 for Side 1), followed by dilated convolutions and upsampling. Finally, the output layer fuses shallow and deep features to localize tampered regions. This nested design allows each side path to impose supervision at its native resolution.

To thoroughly clarify the combination mechanism of side and fused outputs in the loss function, it is essential to understand its parallel and aggregative nature. The total loss function is not a single, internally intertwined complex calculation but rather a simple summation of multiple independently calculated loss values. For each training sample processed by the network, the total loss is effectively the sum of five loss components computed, which is defined as follows:


$$\begin{array}{l} L=\overset{N}{\underset{n=1}{\sum }}w_{side}^{n}l_{side}^{n}+w_{fuse}l_{fuse}. \end{array}$$

For this specific model, the formula fully expands into a direct sum of five components, which can be described as follows:


$$\begin{array}{l} L=\left(w_{side}^{1}\cdot l_{side}^{1}\right)+\left(w_{side}^{2}\cdot l_{side}^{2}\right)+\left(w_{side}^{3}\cdot l_{side}^{3}\right)+\left(w_{side}^{4}\cdot l_{side}^{4}\right)+\left(w_{fuse}\cdot l_{fuse}\right). \end{array}$$

Each of these five loss components is independently calculated using the standard Binary Cross-Entropy (BCE) loss function. For any given prediction map (whether a side output or fused output), its BCE loss is calculated as follows:


$$\begin{array}{l} l=-\overset{\left(H,W\right)}{\underset{\left(r,c\right)}{\sum }}\left[y_{G}lny_{S}+\left(1-y_{G}\right)\left(1-y_{S}\right)\right]. \end{array}$$

This formula measures the discrepancy between the predicted probability and the ground truth on a pixel-by-pixel basis. Here, *y*_*G*_ is the value of the ground-truth mask at pixel (*r, c*) (1 for tampered, 0 for pristine), while *y*_*S*_ is the network's predicted probability that the pixel belongs to a tampered region. This process means the network generates five prediction maps, and each of these five maps is compared against the same single ground-truth map, resulting in five independent BCE loss values.

One of the most strategic decisions in this design is setting all loss term weights ($w_{side}^{n}$ and *w*_*fuse*_) to 1. This embodies an “equally important” strategy, sending a clear signal to the network that making an accurate prediction at every scale is just as important as making the final, precise prediction. This training strategy for an “honest network” prevents the model from “taking shortcuts” during the learning process. It forces the network to avoid being “lazy” and deferring all difficult decisions to the final high-resolution stages.

During backpropagation, because the total loss is a simple sum of the five components, the total gradient is correspondingly a simple sum of the five component gradients:


$$\begin{array}{l} \nabla L=\nabla l_{side}^{1}+\nabla l_{side}^{2}+\nabla l_{side}^{3}+\nabla l_{side}^{4}+\nabla l_{fuse}. \end{array}$$

This process is analogous to having five “teachers” instructing the network simultaneously: one “general teacher” (*l*_*fuse*_) evaluating the final comprehensive performance, while four “specialist teachers” (*l*_*side*_) evaluating performance at different scales. This multi-pronged gradient flow provides short and strong update paths for the deep layers of the network (like the early stages of the encoder), directly and effectively addressing the vanishing gradient problem and compelling the network to learn a more comprehensive and robust feature hierarchy.

### 3.2 Multi-scale feature extraction

**Figure 4:**
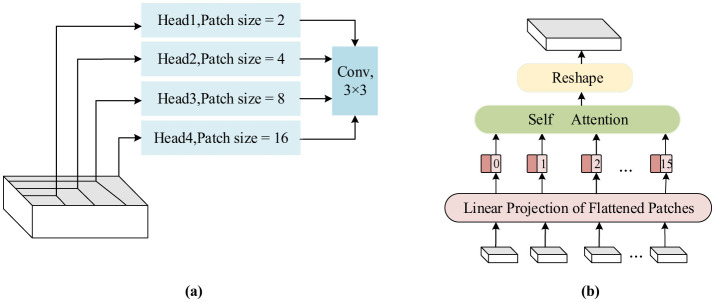
The structure of the multi-scale feature extraction module. **(a)** A diagram of feature extraction using different scales of the header, **(b)** the specific flow of feature extraction using the 4 × 4 scale as an example.

In this model, to further detect tampered regions of different sizes at multiple scales, the multi-scale Transformer is introduced into the network, as shown in Figure 4. It is built based on a Transformer that can be more sensitive to different scales of regions on the feature map and has better global properties than CNNs (Wang et al., 2022). Typically, CNNs suffer from limited receptive fields, and the attention can only be focused on a portion of the region. Therefore, it is often necessary to construct a network by stacking multiple layers. However, the Transformer (Vaswani et al., 2017; Dosovitskiy et al., 2020) allows the modeling of global information more efficiently due to its self-attention mechanism and enhances the network's representation by mapping the feature map to multiple spaces.

In the proposed model, the last layer of the encoding stage is equipped with the multi-scale Transformer to extract multi-scale features. As discussed in the previous section, the output feature map size of the encoding stage is 16 × 16 × 512, which serves as the input to this module. To learn at multiple scales, the feature map is divided into different-sized patches, and different heads of self-attention are calculated. Specifically, it is evenly split into four feature maps along the channel, and four different scales are set: 16 × 16, 8 × 8, 4 × 4, and 2 × 2. The feature map is sampled to obtain different patches at these four scales: 1 × 128 feature maps are obtained using 16 × 16 sampling, 4 × 128 feature maps are obtained using 8 × 8 sampling, 16 × 128 feature maps are obtained using 4 × 4 sampling, and 64 × 128 feature maps are obtained using 2 × 2 sampling. The feature map sequence obtained at each scale is adjusted to a one-dimensional vector sequence, and the feature vector *q* is obtained through linear mapping and concatenation with positional embedding vectors. Similar operations are repeated to obtain feature vectors *k* and *v*. At different scales, self-attention is calculated by using feature vectors *q*, *k*, and *v*, respectively. Finally, the outputs at each scale are reshaped into the original spatial resolution and concatenated together, then sent to the 3 × 3 convolution to obtain the features of the multi-scale Transformer. The use of multi-scale feature encoding improves the localization accuracy of the network, expands the global field of view, and avoids stacking too many layers, which would make the network parameters too numerous.

## 4 Experimental results

This section presents the details of the dataset and experimental setup and provides the experimental results and performance analysis.

### 4.1 Implementation details and evaluation metrics

To train the proposed model, the stochastic gradient descent method was used for optimizing the network, with the learning rate of 0.0005, a batch size of 10, a momentum of 0.9, and a weight decay of 0.0005, which are shown in Table 1. Hyperparameters. The parameters of all layers were initialized using PyTorch's default parameter initialization (Paszke et al., 2017), that is, with normally distributed random numbers. All experiments in the section were conducted on a computer equipped with an NVIDIA RTX 3090 GPU. The evaluation metrics for the network in image splicing tampering localization were precision (P), recall (R), and F1 score. The formulas for calculating these metrics are as follows:


$$\begin{array}{l} P=\frac{TP}{TP+FP},\\ R=\frac{TP}{TP+FN},\\ F1=\frac{2\times P\times R}{P+R}, \end{array}$$

where the pixels of the image are classified as tampered or untampered, the number of tampered pixels correctly detected by the model is *TP*, the number of untampered pixels incorrectly detected is *FN*, and the number of pixels incorrectly detected as tampered is *FP*. Therefore, *P* is the proportion of the number of tampered pixels correctly detected to the number of pixels detected as tampered, and *R* is the proportion of the number of tampered pixels correctly detected to the number of tampered pixels in the image. The *F*1 score is a metric that measures the accuracy of the model, considering both precision and recall of the model. It is the weighted average of model precision and recall.

**Table 1:** Hyperparameters.

**Learning rate**	**Batch size**	**Momentum**	**Weight decay**
0.0005	10	0.9	0.0005

### 4.2 Dataset

Two image tampering datasets, the CASIA (Dong et al., 2013) and COLUMB (Paszke et al., 2017) datasets, were used for evaluation in the experiment. The CASIA dataset includes the CASIAv1 and CASIAv2 datasets and mainly consists of two types of tampering: splicing tampering and copy-move tampering, with the CASIAv2 dataset containing more samples. Therefore, the CASIA dataset in the experiment refers to the CASIAv2 dataset. Splicing tampering images from the CASIA dataset were selected for the experiment, where most of the images were post-processed, such as by compression. In the CASIA dataset, 100 tampered images were randomly selected as the test set. The COLUMB dataset is also a dataset consisting of splicing images. As the dataset only provides the label mask in RGB mode, it was properly processed to obtain a binarized label. Likewise, 44 images were randomly selected from the COLUMB dataset as the test set. The tampered images in the datasets without any data augmentation were called source images and were used for testing. The tampered images with data augmentation using horizontal and vertical flips were used for training, and the image size was resized to 256 × 256. The distributions of the two datasets for the experiments are shown in Table 2.

**Table 2:** Dataset distribution for the training and testing sets.

**Dataset**	**Case**	**CASIA**	**COLUMB**
Training Set	Augmented splicing	2,860	500
	Source image	715	125
Testing Set	Source image	100	44

To ensure a fair and unbiased evaluation of the proposed model's components, all internal ablation experiments (as detailed in Section 4.3.1) were conducted using the exact same training and testing splits described above. For comparisons against other state-of-the-art methods (as detailed in Section 4.3.2), the reported performance metrics for competing models are cited from their respective original publications. As such, the data splits used in those studies may differ from the splits used in this study. This comparison is intended to position the proposed model's performance within the context of existing literature and demonstrate its competitive capabilities.

### 4.3 Experimental results

Details regarding the experiments and performance analysis of the network are provided. The compared methods included traditional methods based on manual design features (Mahdian and Saic, 2009; Ferrara et al., 2012; Ye et al., 2007) and advanced methods based on deep learning (Xiao et al., 2020; Bi et al., 2019; Wei et al., 2021; Huh et al., 2018; Zhao and Tian, 2022).

#### 4.3.1 Ablation experiment

To verify the effectiveness of the network constructed based on U^2^-Net and the multi-scale feature extraction module, the effectiveness of the U^2^-Net-based backbone and multi-scale feature extraction were tested using the CASIA dataset, respectively. The “Base” in Table 3 indicates that only the U^2^-Net-based network was used for training. The “Base-Trans” indicates the use of U^2^-Net and the Transformer encoding module for tampering region localization. As shown in Table 3, the U^2^-Net with the Transformer encoding module achieved better results in all three metrics, while the encoding with multi-scale feature extraction further improved the representational ability of the network, achieving an F1 score 3.1% higher than Base-Trans. It is thus evident that combining U^2^-Net with the multi-scale feature extraction module is effective.

**Table 3:** Comparison of precision, recall, and F1 score on the CASIA dataset.

**Methods**	**Precision**	**Recall**	**F1**
Base	0.775	0.871	0.820
Base-Trans	0.820	0.903	0.860
This model	**0.842**	**0.946**	**0.891**

The bold values denote the best-performing results.

#### 4.3.2 Comparison with other existing methods

To evaluate the tampering localization capability of the network, it was compared with other methods. Table 4 shows the comparison of the proposed model with other methods on the CASIA dataset. The F1 score of the proposed model reached 0.891. Although the proposed model was lower than Mobile-Pspnet (Zhao and Tian, 2022) in terms of precision, the recall of the model was 14.5% higher than that of Mobile-Pspnet. In addition, on the CASIA dataset, the F1 score of the proposed model outperformed other methods. It was 2.5% higher than MCNL-Net (Wei et al., 2021), which had the best F1 score performance among these methods. Although MCNL-Net utilizes residual propagation and feedback to enhance the learning capability of the CNN and uses the BAM attention mechanism (Park et al., 2018), its ability to capture global contextual information in images is still limited. The proposed model uses multi-scale feature extraction, which further extends the global views of the network and contributes to better learning of the features by the network.

**Table 4:** Performance comparison of the proposed model with other methods on the CASIA dataset.

**Methods**	**Precision**	**Recall**	**F1**
CFA (Ferrara et al., 2012)	0.057	0.846	0.108
DCT (Ye et al., 2007)	0.349	0.871	0.498
NOI (Mahdian and Saic, 2009)	0.079	0.088	0.083
C2R-Net (Xiao et al., 2020)	0.417	0.424	0.420
FCN (Long et al., 2015)	0.509	0.173	0.259
MCNL-Net (Wei et al., 2021)	0.909	0.828	0.866
RRU-Net (Bi et al., 2019)	0.848	0.834	0.841
Mobile-Pspnet (Zhao and Tian, 2022)	**0.910**	0.801	0.832
This work	0.842	**0.946**	**0.891**

The bold values denote the best-performing results.

Table 5 shows the comparison of the proposed model with other methods on the COLUMB dataset, and the proposed model was slightly lower than Mobile-Pspnet in terms of the F1 score. Considering that the splicing tampered images in the COLUMB dataset originate from different cameras, both captured and synthesized, the noise caused by the different cameras could potentially affect the detection capability of the proposed model. However, the proposed model's F1 score was still 9.2% higher than that of MCNL-Net. As shown in Tables 4, 5, the deep learning-based approach for image tampering detection outperformed the traditional detection methods. The proposed model achieved the best results on the CASIA dataset and outperformed most of the methods on the COLUMB dataset among the deep learning-based methods.

**Table 5:** Performance comparison of the proposed model with other methods on the COLUMB dataset.

**Methods**	**Precision**	**Recall**	**F1**
CFA (Ferrara et al., 2012)	0.574	0.469	0.517
DCT (Ye et al., 2007)	0.365	0.633	0.463
NOI (Mahdian and Saic, 2009)	0.321	0.015	0.028
DF-Net (Huh et al., 2018)	0.528	0.468	0.496
C2R-Net (Xiao et al., 2020)	0.576	0.097	0.166
FCN (Long et al., 2015)	0.859	0.443	0.584
MCNL-Net (Wei et al., 2021)	0.839	0.715	0.772
RRU-Net (Bi et al., 2019)	0.961	0.873	**0.915**
Mobile-Pspnet (Zhao and Tian, 2022)	**0.964**	0.852	0.881
This work	0.764	**0.994**	0.864

The bold values denote the best-performing results.

A closer examination of Tables 4, 5 reveals an important performance nuance: The proposed model achieved the state-of-the-art result on the CASIA dataset but was outperformed by models such as RRU-Net and Mobile-Pspnet on the smaller COLUMB dataset. This discrepancy is unlikely due to simple overfitting but rather stems from an interplay between this model's architectural complexity and the distinct characteristics of each dataset.

A primary factor to consider is the substantial discrepancy in the scale of the employed datasets. The CASIA training set was more than five times larger than the COLUMB set (2,860 vs. 500 augmented images). The proposed network, featuring a deep U^2^-Net backbone and a multi-scale Transformer, possesses a high learning capacity. This complexity is an advantage in large datasets such as the CASIA dataset, as it allows the model to learn highly generalizable features of splicing tampering. It is worth noting that different types of models may exhibit their respective strengths depending on the scale of the dataset. The model was designed with complex architecture to learn intricate feature patterns from large-scale, diverse data. Consequently, when applied to a scenario with relatively limited data, such as the COLUMB dataset, its performance may not be fully optimized. Concurrently, some models whose inductive biases are more aligned with small-sample learning tasks can achieve a more robust fit on a limited data distribution by virtue of their structural advantages.

Therefore, the performance variation reflects a combined effect: the proposed model excels on large-scale datasets where its high capacity is an asset but shows relative sensitivity to the specific type of artifact heterogeneity found in the smaller COLUMB dataset.

#### 4.3.3 Qualitative results

**Figure 5:**
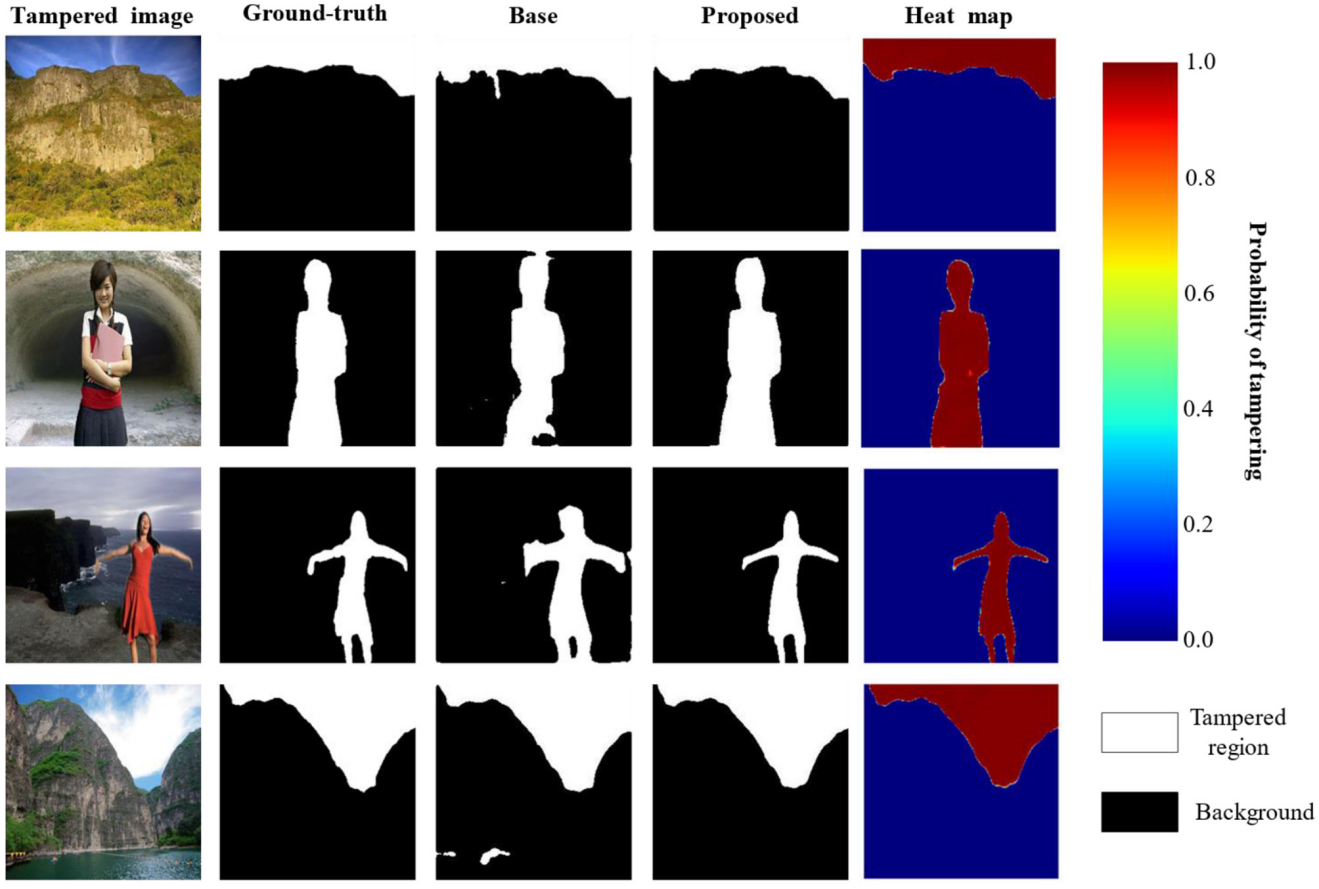
Results of image tampering region localization (reproduced from the CASIA dataset, Dong et al., 2013).

The qualitative results for tampering localization by the proposed model are presented in Figure 5, which primarily shows the comparison of the baseline model (Base) and the proposed model. The figure's columns, respectively, display the tampered image, the ground-truth mask, the prediction from the base model, and the prediction from the proposed model. Furthermore, the heat map column, along with its corresponding color bar, visualizes the model's prediction probability, where the color transition from blue to red indicates an increasing probability from 0.0 to 1.0. A clear comparison revealed the superiority of the proposed model, as it was more effective at detecting the edges and ensuring the completeness of the spliced regions, which is particularly evident in the second and third rows. In contrast, the base model suffered from significant false negatives, failing to identify large portions of the actual tampered regions in all four examples, resulting in large black holes and missing sections. This demonstrates the inadequacy of its network's capability to form a complete and global feature representation. The performance of the proposed model was substantially better, a result attributed to its utilization of a multi-scale feature extraction module that enhances the network's global perspective and effectively strengthens the integrity of its feature representations.

#### 4.3.4 Robustness

**Figure 6:**
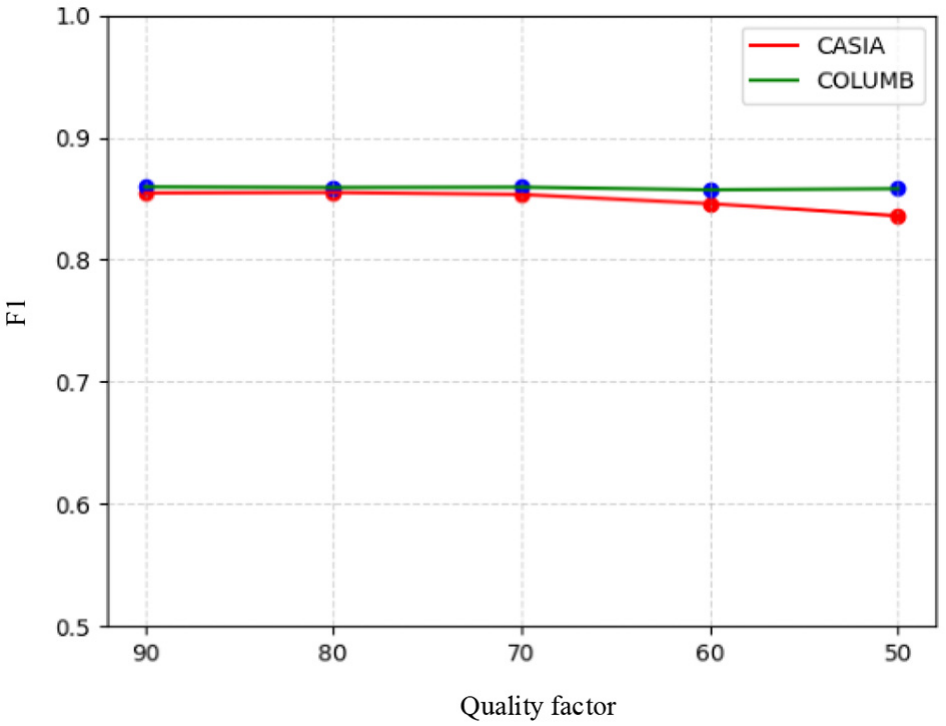
F1 scores based on the compression of different quality factors.

**Figure 7:**
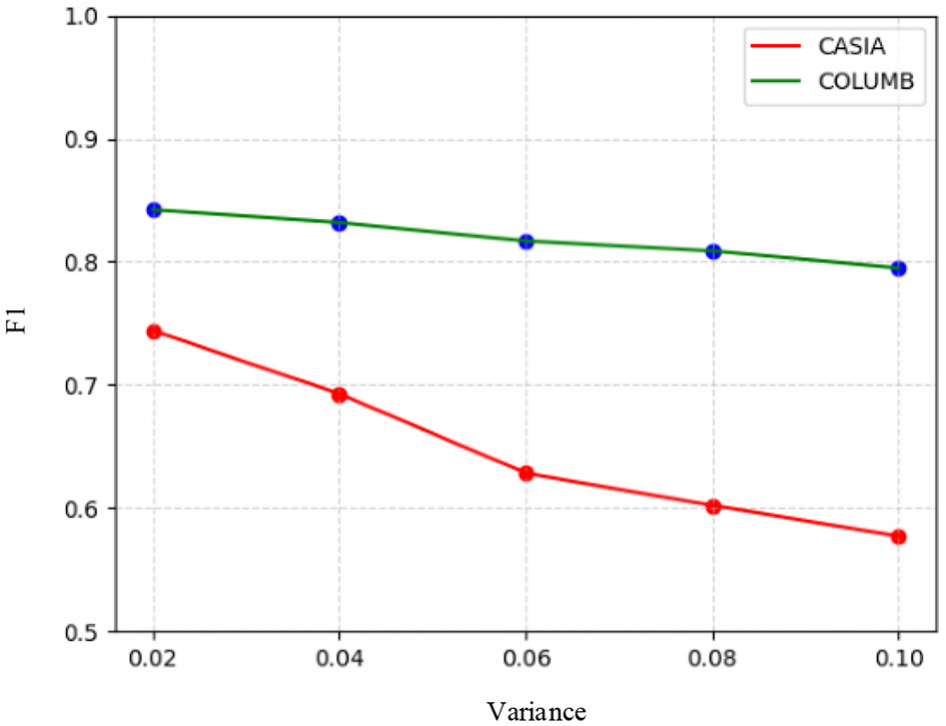
F1 scores based on Gaussian white noise with different variances.

To evaluate the robustness of the proposed model, it was tested on the test sets of the CASIA and COLUMB datasets, where the post-processing operations used for testing included JPEG compression and the addition of Gaussian white noise. Specifically, JPEG quality factors of 90, 80, 70, 60, and 50 were used for compression on the test sets of the two datasets, respectively, followed by splicing tampering localization using the model. Similarly, Gaussian white noise with variances of 0.02, 0.04, 0.06, 0.08, and 0.1 was processed on the test sets of the two datasets, respectively, and splicing tampering localization was performed using the model. The F1 scores of the model in response to JPEG compression and Gaussian white noise are shown in Figures 6, 7. It can be seen that the overall change in the F1 scores of the model was not significant, despite the use of compression with different quality factors on the datasets. On the other hand, the F1 scores obtained by the model for different intensities of noise detection showed a decreasing trend across both datasets. Therefore, the model demonstrated good robustness against JPEG compression attacks. However, it was somewhat affected by high-intensity noise interference.

Furthermore, the datasets diverge significantly in terms of the specific types of artifacts they contain. The CASIA dataset primarily features images with post-processing artifacts such as JPEG compression. The model's strength—its ability to fuse local and global multi-scale information—allows it to look beyond uniform compression artifacts to identify deeper structural and semantic inconsistencies. In contrast, the COLUMB dataset is characterized by noise heterogeneity arising from the use of different cameras for synthesis. As speculated, the model's focus on structural context might make it more sensitive to inconsistent, high-frequency noise patterns, which could disrupt feature extraction. Models such as RRU-Net or Mobile-Pspnet might be less affected by this specific type of noise or may be implicitly tuned to different artifact cues that are more prevalent in the COLUMB dataset, explaining their strong performance. It is somewhat difficult to identify other transformative operations such as blurring and scaling, which fundamentally degrade the forensic clues the model relies on. Blurring operations, acting as a low-pass filter, would likely pose a significant challenge by directly removing the high-frequency components where tampering artifacts such as sharp splicing boundaries and noise inconsistencies reside, thereby eliminating the primary evidence for detection. Similarly, resizing disrupts the image's integrity by introducing new, uniform pixel patterns across the entire canvas via interpolation. This process can obscure or corrupt the original noise patterns and pixel correlations at the splice boundary, with downscaling being particularly detrimental due to the associated information loss that can erase subtle artifacts.

Theoretically, comparing the model's robustness with that of other state-of-the-art methods involves assessing the reliance of different architecture on specific types of artifacts. For instance, dual-stream networks that heavily leverage frequency-domain analysis might be more sensitive to JPEG artifacts but could exhibit different vulnerabilities to noise attacks compared to the proposed model. In contrast, the core strength of this model lies in its fusion of U^2^-Net's nested multi-scale structure with the Transformer's global context modeling. This design enables it to perceive not only local high-frequency details but also global structural and semantic inconsistencies. Therefore, this study posits that the model may exhibit a relative advantage against attacks that disrupt local details while preserving some global structures (e.g., moderate compression). A comprehensive robustness benchmark would be invaluable for validating these theoretical advantages and disadvantages across different architectural designs.

#### 4.3.5 Discussion

To comprehensively evaluate the proposed model, benchmarking against diverse state-of-the-art methods is essential. For instance, IFE-Net (Su et al., 2025) is an integrated dual-stream network that enhances RGB and noise features through a feedback-enhanced ASPP module and a CBAM attention module, respectively, while utilizing edge supervision to refine localization. M2BG-Net (Jiang et al., 2025) introduces a multi-modality boundary-guided network that jointly optimizes RGB, high-frequency features, and boundary artifacts through dedicated modules to improve generalization for image manipulation localization. MSNP-Net (Zhang et al., 2024) presents a dual-branch progressive network that uses a multi-scale noise branch to guide a multi-resolution feature branch and progressively fuses features to achieve accurate image splicing detection. The adaptive multi-feature filtration method (Hou et al., 2024) extracts noise artifact and ELA-weighted features, represents them as a point cloud for feature filtration, and uses Alpha-shape trend connectivity to aggregate them for final localization. This method (Qin et al.) is distinct as it utilizes single-stream RGB input architecture with a nested U-Net (U^2^-Net) backbone, unlike the dual-stream (e.g., RGB and noise/frequency) designs in IFE-Net (Su et al., 2025), M2BG-Net (Jiang et al., 2025), and MSNP-Net (Zhang et al., 2024). Its primary innovation lies in the hierarchical multi-scale feature fusion strategy, combining the rich local context from nested RSU blocks with explicit long-range global dependencies modeled by a multi-scale Transformer module inserted between the encoder and decoder. This deep learning approach, which also employs a deep supervision strategy, fundamentally differs from the non-deep learning, feature-engineering pipeline proposed by Hou et al. (2024).

The two-branch architecture recently proposed by Luo et al. (2024) represents a successful alternative strategy. By simultaneously analyzing an image's spatial (RGB) and frequency information and fusing them with an attention mechanism (CBAM), their model achieves excellent performance on datasets such as NIST16. This explicit use of frequency-domain clues might give their model a distinct advantage in detecting specific high-frequency artifacts introduced by operations such as JPEG compression.

While the proposed model in this study also achieves competitive results (F1 score of 0.891 on the CASIA dataset), its strengths stem from a different source. Through a deeply supervised U^2^-Net and a multi-scale Transformer, it achieves powerful global contextual modeling. This allows the proposed model to excel at ensuring the completeness of the detected tampered regions and avoiding large-scale false negatives, a fact supported by qualitative results (Figure 4). It is important to note that a direct numerical comparison of F1 scores is not possible due to differences in dataset partitioning and test set selection between the two studies (e.g., 100 images from the CASIA dataset were tested in the experiment, whereas Luo et al., tested on 921 images from the CASIAv1 dataset). In conclusion, the two approaches represent two different yet equally valuable technical paths for tackling the complex problem of image tampering localization: one focusing on multi-modal information fusion and the other on deep, multi-scale representation within a single modality.

Significant discrepancies exist between this study and the compared literature regarding the datasets used, specific partitioning methods, and performance evaluation metrics. For example, the comparative studies were evaluated on test sets not covered in this study, such as NIST16, Coverage, and IMD2020. In addition, there are differences in metrics, with the M2BG-Net (Jiang et al., 2025) study employing the Matthews correlation coefficient (MCC). Given these fundamental differences in training paradigms, dataset selection, data partitioning, and evaluation metrics, a direct numerical comparison of performance indicators could lead to an inequitable assessment.

## 5 Conclusion

In this study, a multi-scale, deeply supervised network-based model is proposed for image splicing tamper region localization. The model employs U^2^-Net as the backbone network. Deep supervision is applied to extensively train the network and enhance feature extraction in the shallow layer of the network. In addition, the combination with multi-scale feature extraction enables the model to have multiple perceptual fields, which improves the model's ability to capture global information. Experiments were performed on two public datasets, with a comparison to advanced image splicing tampering methods. The experimental results indicated that the proposed model achieved good results and outperformed most localization methods. Although the model is relatively better than many other methods in splicing localization, its generalization ability still needs to be further improved, especially since its performance is more limited when dealing with small datasets. The inability of small datasets to cover sufficient diversity of situations and the fact that the model does not have enough samples to learn enough important tampering features may affect the generalization ability of the model.

Subsequent research will focus on several key areas to build upon the current study. To address the model's limited performance on smaller datasets, future research will explore advanced data augmentation techniques, such as using generative adversarial networks (GANs) for data extension, and investigate more effective loss functions to improve localization precision.

Furthermore, a primary focus will be on conducting a more comprehensive and rigorous robustness evaluation. This should involve testing the model's resilience against a wider array of common transformations not covered in this study, such as image resizing and blurring, which are known to fundamentally degrade high-frequency forensic clues. Furthermore, these robustness tests should be performed in a direct comparative framework, benchmarking the proposed model against other state-of-the-art methods under identical attack conditions to provide a more thorough and equitable assessment of its capabilities. Finally, future architectural enhancements will specifically aim to improve the model's resilience to high-intensity, unstructured noise, as the analysis in this study identified this as a significant challenge that can mask subtle tampering artifacts.

## Data Availability

The original contributions presented in the study are included in the article/supplementary material, further inquiries can be directed to the corresponding author.
